# Three *de novo* variants in *KMT2A* (*MLL*) identified by whole exome sequencing in patients with Wiedemann–Steiner syndrome

**DOI:** 10.1002/mgg3.1798

**Published:** 2021-09-01

**Authors:** Sukun Luo, Bo Bi, Wenqian Zhang, Rui Zhou, Wei Chen, Peiwei Zhao, Yufeng Huang, Li Yuan, Xuelian He

**Affiliations:** ^1^ Precision Medical Laboratory Wuhan Children's Hospital (Wuhan Maternal and Child Healthcare Hospital) Tongji Medical College Huazhong University of Science & Technology Wuhan China; ^2^ Rehabilitation Department Wuhan Children's Hospital (Wuhan Maternal and Child Healthcare Hospital) Tongji Medical College Huazhong University of Science & Technology Wuhan China; ^3^ BGI Genomics BGI‐Shenzhen Shenzhen China; ^4^ BGI‐Wuhan Clinical Laboratories BGI‐Shenzhen Wuhan China; ^5^ Department of Biology University of Copenhagen Copenhagen Denmark; ^6^ State Key Laboratory of Agricultural Microbiology College of Life Science and Technology Huazhong Agricultural University Wuhan China; ^7^ Ultrasonography Department Wuhan Children's Hospital (Wuhan Maternal and Child Healthcare Hospital) Tongji Medical College Huazhong University of Science & Technology Wuhan China

**Keywords:** *de novo* variant, endocardial fibroelastosis, *KMT2A*, neurodevelopment delay, Wiedemann–Steiner syndrome

## Abstract

**Background:**

Wiedemann–Steiner syndrome (WSS) is an autosomal dominant disorder characterized by short stature, hypertrichosis, intellectual disability, developmental delay, along with facial dysmorphism. WSS patients exhibit great phenotypic heterogeneities. Some variants in *KMT2A* (*MLL*) gene have been identified as the cause of WSS.

**Methods:**

Whole exome sequencing on the probands followed by Sanger sequencing validations in the family were applied to determine genetic variants. In silico analyses were used for predicting potential effects of the variants.

**Results:**

We identified three novel *de novo* heterozygous variants: c.883A>T (p.Lys295*), c.4171C>T (p.Gln1391*), and c.3499T>C (p.Cys1167Arg), in *KMT2A* gene from three unrelated Chinese WSS patients. According to the American College of Medical Genetics and Genomics (ACMG) guidelines, these three variants were classified as pathogenic, pathogenic and likely pathogenic variant, respectively. By reviewing all the available cases with same mutated KMT2A regions as the three patients had, we found that in addition to the representative symptoms, our patients exhibited some sporadically observed symptoms, such as severe ophthalmological symptoms, endocardial fibroelastosis, cytomegalovirus infection, and feet eversion. We also revealed that variants in different KMT2A regions contribute to the phenotypic heterogeneity of WSS, highlighting challenges in the diagnosis of syndromic disorders spanning a broad phenotypic spectrum.

**Conclusion:**

Our study would aid in further broadening our knowledge about the genotype–phenotype correlation of WSS.

## Graphical abstract

Endocardial fibroelastosis and feet eversion are newly identified features in Wiedemann–Steiner syndrome. Three novel do novo variants identified in this study help to further associate genotype with phenotype in WSS.

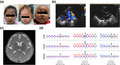

## 1 INTRODUCTION

Wiedemann–Steiner Syndrome (WSS, OMIM #605130) is a very rare multiple congenital anomaly syndrome that shows autosomal dominant inheritance. WSS is characterized by developmental delay, intellectual disability, short stature, hypertrichosis along with distinctive facial appearance (Jones et al., 2012). The prevalence of WSS is estimated to be less than 1 in 1,000,000 (www.orpha.net, ORPHA: 319182). It was firstly described by Wiedemann et al. (1989). Thereafter, a variety of WSS cases were reported worldwide, but they exhibited great phenotypic heterogeneities (Baer et al., 2018; Grangeia et al., 2020; Jones et al., 2012; Li et al., 2018).

WSS is caused by variants in the *KMT2A* (Lysine Methyltransferase 2A, also known as *MLL*, OMIM #159555) gene (Jones et al., 2012), which is also implicated in the disease of leukemia (Meyer et al., 2006; Peterson et al., 2018). To date, 131 *KMT2A* variants with clinical significances have been identified according to HGMD Professional database (version 2019.1; http://www.hgmd.cf.ac.uk/ac/index.php), and the majorities are truncating variants (i.e., frame‐shift, nonsense, and splicing variants; 88/131; 67.18%), followed by missense variants (37/131; 28.24%). Among these variants, 66 are associated with WSS.

*KMT2A* encodes a transcriptional coactivator KMT2A (also named MLL), which is crucial for gene expression regulation through its function of histone methyltransferase during early development and hematopoiesis (Ronan et al., 2013). KMT2A is a large protein of 3,969 amino acid residues, and it is a structurally complex protein composed of several conserved functional domains, including three DNA‐binding AT‐hook at the N terminus, a cysteine‐rich CXXC domain, four plant homeodomain (PHD) finger motifs, a bromodomain domain (BD), a transactivation domain (TAD), a FY (phenylalanine tyrosine)‐rich N terminus (FYRN) motif, a WDR5 interaction (Win) motif, and finally a catalytic SET (Suppressor of variegation, Enhancer of zeste, Trithorax) domain at the C‐terminus. Among these domains, the SET domain is responsible for histone H3 lysine 4 (H3K4) methyltransferase activity which mediates epigenetic transcriptional activation (Milne et al., 2002). The AT‐hook and the CXXC domain are capable of binding AT‐rich DNA and nonmethylated CpG‐containing DNA, respectively; they are essential for fusing KMT2A to other proteins, such as nuclear transcription factors, transcriptional coactivators, chromatin remodeling protein, as well as cytoplasm heterologous proteins (Liu & Min, 2019; Zeleznik‐Le et al., 1994). These fusion proteins are then implicated in various cell‐transforming processes. The nuclear‐targeting sequences located between AT‐hook and CXXC domains contribute to the nuclear localization of KMT2A and its fusion proteins (Yano et al., 1997). Previous studies reported that patients with variants in the CXXC domain may display more severe neuro‐phenotypes (Lebrun et al., 2018; Li et al., 2018; Min Ko et al., 2017). However, more studies would be needed to further delineate the genotype–phenotype correlation of WSS.

In this study, we performed whole exome sequencing on three unrelated Chinese WSS patients, and identified two novel truncating variants and one novel missense variant in *KMT2A*. We elaborated clinical and genetic features of these cases, and compared them with available WSS cases. We commented on how variants locating in different KMT2A domains contribute to the phenotypic heterogeneity. Our study further expanded the mutation spectrum of *KMT2A* and provided abundant evidences on the genotype–phenotype correlation of WSS.

## 2 MATERIALS AND METHODS

Our study was approved by the ethics committee and review board of Wuhan Children's Hospital (Wuhan Maternal and Child Healthcare Hospital). After obtaining written informed consents from patients’ parents, genomic DNA were extracted from the whole blood of three patients and their parents. Trio‐based whole exome sequencing was performed on Illumina HiSeq2000 sequencer according to the manufacturer's instructions. The exonic coverage at 80‐fold sequencing depth was 99.8%. The variants of interests were confirmed by Sanger sequencing in patients and their parents. The reference sequence used for *KMT2A* was NM_001197104.2.

## 3 RESULTS

### 3.1 Case report

#### 3.1.1 Patient 1 (P1)

**FIGURE 1:**
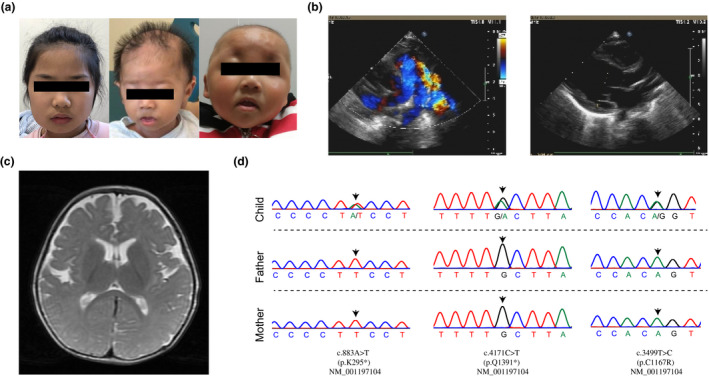
Clinical features and *KMT2A* variants of three WSS patients. (a) P1 had mild facial asymmetry, epicanthus tarsalis accompanied with severe ptosis of upper eyelid and small palpebral fissures, as well as astigmatism (left). P2 had sparse scalp hair, bilateral ptosis, mild hypertelorism, down‐slanting palpebral fissure, low‐set ears, long eyelashes, long philtrum, hypertrichosis, thin upper lip, and micrognathia (middle). P3 had prominent forehead, hypertelorism, wide nasal bridge, and low‐set ears (right). (b) Echocardiography indicated the likelihood of endocardial fibroelastosis, patent ductus arteriosus and patent foramen ovale in P2. (c) Brain magnetic resonance imaging revealed enlarged bilateral fissure and anterior longitudinal division. (d) Sanger sequencing validated three *de novo* variants in *KMT2A* in P1 (left), P2 (middle), and P3 (right).

The first patient was a 10‐year‐old girl with a history of short stature, growth retardation, intellectual disability, hirsutism, and ocular anomaly. She was born to non‐consanguineous Chinese parents via vaginal delivery at 38 weeks, with a birth weight of 3.1 kg. No obvious intra‐uterine growth restriction was observed by antenatal ultrasound scanning. She was noticed to have growth retardation at 1 year of age. At 4 years old, she was referred to our endocrinology department for comprehensive evaluations. Physical exam suggested that she had no obvious facial dysmorphism (Figure 1a left), while her height was 84.8 cm (<−3 SD) and weight was 23 kg (>2 SD). Basal hormonal evaluation revealed normal levels of T3, T4, TSH (thyroid stimulating hormone), CORT (8 am and 8 pm), ACTH (adrenocorticotropic hormone), and IGFBP3 (insulin‐like growth factor binding protein), but decreased level of IGF‐1 (insulin‐like growth factor‐1; 33.2 ng/ml, reference range >55 ng/ml), which suggested IGF‐1 deficiency. Glucagon stimulation testing showed peak growth hormone (GH) level was 10.5 ng/ml (reference range >10 ng/ml), indicating no obvious GH deficiency. A hand x‐ray showed that her bone age was only 2.5 years. Brain MRI (Magnetic Resonance Imaging) identified increased pituitarium. At the age of 10 years, she came to our rehabilitation department for re‐evaluation, since she exhibited symptoms of poor learning ability in mathematics, severe sensory integration dysfunction, as well as defective communication and socialization skills. She presented mild facial asymmetry, epicanthus tarsalis accompanied with severe ptosis of the upper eyelid and small palpebral fissures, as well as serious ocular abnormality of astigmatism (Johnson, 1978). In particular, her height (118 cm) and weight (23 kg) were more than 3 SD below the mean values for her age.

#### 3.1.2 Patient 2 (P2)

The second patient was a 5‐month‐old girl with a history of congenital cardiac malformation, recurrent infections, distinctive facial features, global developmental delay, and muscular hypotonia. She was born at the 37th week of gestation by vaginal delivery due to premature rupture of the amniotic fluid, with birth weight of 2.8 kg (<−1 SD) and birth length of 47 cm (<−1 SD). She was the first child of healthy, non‐consanguineous parents. Her mother had minor vaginal bleeding and no remarkable history during the prenatal and neonatal periods. At the first month and 22 days, she was referred to our pneumology department due to severe paroxysmal cough and failure to thrive. On examination, her length was 51 cm (<−2 SD), weight 3.5 kg (<−2 SD). She had very low‐pitched crying and poor responses to various actions. Cytomegalo virus (CMV) test suggested positive cytomegalovirus infection (CMV‐IgG = 3.609 g/L, and CMV‐IgM = 7.2243 g/L). Serum immune function testing revealed slightly increased level of IgA (0.36 g/L, reference range 0.14–0.34 g/L) as well as normal levels of C3, C4, IgG, and IgM. Echocardiographic evaluation showed increased left ventricular internal diameter (30 mm, reference range 11.82 ± 1.15 mm for 3‐month‐old infant; Deng et al., 2006), increased left endocardium, enlarged pulmonary artery, and some left‐to‐right shunts, indicating the likelihood of endocardial fibroelastosis, patent ductus arteriosus and patent foramen ovale (Figure 1b). Consequently, she was transferred for cardiac surgery of patent ductus arteriosus ligation at 3.5 months. Re‐examination after the surgery did not identify residual shunting at the arterial level. At 5 months, she was referred for re‐evaluation due to failure of holding up her head on her own, which was expected by the time of 3 months old. At this age, her height was 59 cm (<−2 SD) and weight 5 kg (<−3 SD). She had malnourished and dysmorphism face, sparse scalp hair, bilateral ptosis, mild hypertelorism, down‐slanted palpebral fissure, low‐set ears, long eyelashes, long philtrum, hypertrichosis on her arms and legs, thin upper lip, micrognathia, as well as small hands and feet (Figure 1a middle). She was unable to roll over or raise head, and her lower limbs could not bear the body weight when she stood with help. Her developmental assessments on motor skills, learning behaviors, language, and social adaptation using the Gesell Developmental Schedule indicated global developmental delay. Brain MRI showed enlargements in both bilateral fissure and anterior longitudinal division (Figure 1c).

#### 3.1.3 Patient 3 (P3)

The third patient was a 20‐month‐old girl with a history of muscular hypotonia, global developmental delay, skeletal anomalies, and unusual face. She was the second child of healthy, non‐consanguineous parents, and had a healthy elder brother. Her mother had a remarkable pregnancy history of two spontaneous abortions and two induced abortions. During the prenatal stage of this girl, her mother was diagnosed with an increased level of amniotic fluid volume by ultrasound scanning. She was born via vaginal delivery at 37 weeks of gestation due to premature rupture of the amniotic fluid, with weight of 2.65 kg (<−1 SD) and height of 47 cm (<−1 SD). At birth, she had no distinctive facial dysmorphism, but hirsutism was found on her ears, which fell off after one month. She was able to raise her head at 6 months and sit at 10 months. At 20‐month‐old, she was yet unable to crawl or walk independently, and referred to the hospital. She had bilateral foot eversion. The neurological examination indicated that four limbs had generalized muscle hypotonia and generalized weakness with Medical Research Council (MRC) grade 4. Her height was 69 cm (<−3 SD) and weight was 10 kg (<−2 SD). She had some distinctive dysmorphic facial features, including prominent forehead, hypertelorism, wide nasal bridge, and low‐set ears (Figure 1a, right). Development quotient (DQ) results of her Gesell development examination were 22 in adaption, 27 in gross motor, 22 in fine motor, 31 in speech, and 27 in personal‐social, indicating developmental delay. Her cognitive function assessment showed impaired cognitive capacity of only 5–7 months old. The Activity of Daily Living assessment revealed poor activity (score = 7.5). Brain MRI revealed enlargement of bilateral frontal and temporal subarachnoid space, as well as choroid plexus cysts.

### 3.2 Whole exome sequencing revealed three novel variants

After performing trio‐based whole exome sequencing followed by Sanger sequencing confirmation, we identified three *de novo* heterozygous variants in the *KMT2A* gene NC_000011.10 (NM_001197104.2): c.883A>T (p.Lys295*) in patient 1, NC_000011.10 (NM_001197104.2): c.4171C>T (p.Gln1391*) in patient 2, and NC_000011.10 (NM_001197104.2): c.3499T>C (p.Cys1167Arg) in patient 3 (Figure 1d, Table 1). According to the ACMG guidelines, these variants were classified as pathogenic, pathogenic and likely pathogenic, respectively. The nonsense variant p.Lys295* occurred in exon 3 of *KMT2A* gene and located in the AT‐hook domain of KMT2A protein. The other nonsense variant p.Gln1391* in exon 9 of *KMT2A* could introduce a premature termination in an intermediate region between CXXC and PHD domains of KMT2A protein. The missense variant p.Cys1167Arg located in exon 5 of *KMT2A*, which encoded the CXXC domain of KMT2A protein. None of the three variants had been reported previously. The three variants were extremely rare mutations in Chinese population and all were absent in the ChinaMap database (http://www.mbiobank.com/).

**TABLE 1:** Clinical and molecular features of Wiedemann–Steiner Syndrome patients with causative variants in different regions of KMT2A

	AT‐hook domain				Intermediate region between CXXC and PHD domain							CXXC domain						
	P1	Baer 2018 (P14)	Li 2018 (P13)	Miyake 2016 (P4)	P2	Baer 2018 (P20)	Baer 2018 (P12)	Ber 2018 (P16)	Li 2018 (P1)	Li 2018 (P11)	Strom 2014 (P2)	P3	Lebrun 2018 (P1)	Stellacci 2016 (P1)	Min 2017 (P5)	Baer 2018 (P26)	Lebrun 2018 (P2)	

Sex	F	F	F	F	F	M	M	M	F	F	M	F	M	M	M	M	M	
Gestation (weeks)	38	38	35+5	ND	37	39	36+3	38	35	38+5	38	37	ND	38	Full term	39	ND	
Current height (cm)/weight (kg)	<−3 SD/<−3 SD	2 SD/0.4 SD	−6.25 SD/−4.13 SD	ND	<−2 SD/<−3 SD	−1.4 SD/−2.6 SD’	3.2 SD/−0.5 SD	−0.6 SD/−0.3 SD’	−1.75 SD/−2.30 SD	−3.28 SD/−2.75 SD	−3 SD/−3 SD	<−3 SD/<−2 SD	<−3 SD/<−3 SD	−6 SD/−4 SD	−2.26 SD/−1.09 SD	−2.1 SD/−3.9 SD	−3 SD/−2 SD	
Nucleotide change^a^	c.883A>T	c.654_679delinsT	c.901C>T	c.838C>A	c.4171C>T	c.3895_3896delTC	c.4012delG	c.4032delG	c.3837delT	c.4061delC	c.4086+1G>A	c.3499T>C	c.3460C>T	c.3481T>G	c.3503G>A	c.3542G>A	c.3581G>A	
Amino Change	Lys295*	Glu219Leufs*27	Arg301*	Pro280Thr	Gln1391*	Ser1299Profs*26	Gly1338Valfs*18	Val1347Trpfs*9	Pro1281Leufs*75	Pro1354Leufs*2	−	Cys1167Arg	Arg1154Trp	Cys1161Gly	Gly1168Asp	Gly1181Asp	Cys1194Tyr	
Craniofacial features																		
Microcephaly	−	−	−	ND	−	−	−	−	+	−	−	−2 SD	−3 SD	+	−	ND	−1 SD	
Prominent forehead	−	−	+	ND	+	−	ND	ND	+	+	+	+	ND	−	+	ND	ND	
Hypertelorism	+	−	+	ND	−	−	+	+	+	+	+	+	+	+	+	−	+	
Down‐turned palpebral fissures	−	−	+	ND	+	ND	+	+	+	+	+	+	ND	+	+	−	ND	
Small palpebral fissures	−	ND	ND	ND	+	+	+	+	ND	ND	+	−	+	+	ND	+	+	
Wide nasal bridge	+	−	−	ND	+	−	−	+	+	+	+	+	+	+	+	−	+	
Depressed nasal bridge	−	ND	−	ND	+	+	ND	ND	+	+	+	+	ND	−	−	ND	ND	
Long philtrum	−	−	+	ND	+	−	+	+	−	+	ND	+	ND	−	+	+	ND	
Thin upper lip	−	−	−	ND	+	+	+	+	+	+	+	−	+	+	+	+	+	
Low set ears	−	+	−	ND	+	ND	−	+	−	−	ND	+	+	−	−	−	+	
Down‐turned corners of the mouth	−	−	+	ND	+	−	ND	ND	+	+	−	+	+	ND	+	ND	+	
Micrognathia	−	−	+	ND	+	−	ND	ND	−	−	+	ND	ND	−	+	ND	ND	
Skeletal anomalies																		
Muscular hypotonia	ND	−	ND	ND	+	ND	ND	−	ND	ND	+	+	ND	+	ND	+	ND	
Advanced bone age	−	ND	ND	ND	ND	ND	ND	+	ND	−	ND	ND	ND	−	+	+	ND	
Delayed bone age	+	ND	ND	ND	ND	−	ND	−	ND	+	ND	ND	ND	ND	−	−	ND	
Small hands and feet	−	ND	−	ND	+	−	ND	+	−	+	+	ND	+	+	−	ND	Larges and swollen	
Hairiness																		
Thick hair	−	−	+	ND	−	ND	ND	ND	+	+	+	ND	ND	+	−	ND	ND	
Thick eyebrows	−	+	−	ND	−	+	+	−	−	+	+	−	+	+	−	+	+	
Arched eyebrows	−	−	−	ND	−	ND	ND	ND	−	+	−	−	ND	−	+	ND	ND	
Long eyelashes	−	−	−	ND	+	+	+	+	+	+	+	−	−	+	+	+	+	
Low hair line	−	−	−	ND	−	ND	ND	ND	+	+	−	−	ND	+	+	ND	ND	
Hypertrichosis	+	−	Mild	ND	+	+	+	+	Mild	Mild	−	+	+	+	+	+	+	
Neurodevelopmental problem																		
Epilepsy	−	−	−	ND	−	+	−	−	−	−	−	−	+, generalized, tonic clonic	+	ND	−	−	
Developmental delay	+	−	+	ND	+	+	+	+	ND	+	+	+	ND	+	+	+	ND	
Language delay	−	−	Too young	ND	Too young	ND	ND	ND	+	Too young	+	+	Lack of language (tongue dystonia)	+	+	+	Two words	
Intellectual disability	Mild	Mild	+	ND	+	Severe	Moderate	Moderate	Mild	+	ND	Severe	Profound	+	+	Severe	Profound	
Aggressive behavior	−	ND	+	ND	−	−	−	+	−	−	ND	−	Hyperactivity	+	+	−	−	
MRI	Increased pituitarium	−	−	ND	Widened bilateral fissure and anterior longitudinal division	Partial CC, agenesis	−	−	ND	ND	ND	Enlarged subarachnoid, choroid plexus cysts	Chiari I	ND	ND	ND	−	
Organic problems																		
Hyperopia	−	−	Too young	ND	ND	−	+	−	−	−	Microphthalmia	ND	ND	ND	−	−	ND	
Astigmatism	+	−	Too young	ND	ND	−	−	−	−	−	Microphthalmia	ND	ND	ND	ND	−	ND	
Feeding difficulties	−	−	+	ND	+	+	+	+	+	−	ND	+	+	+	+	+	+	

^a^ The genomic reference sequence used for *KMT2A* is NM_001197104.2.

### 3.3 Review on the phenotypes associated with KMT2A variants in three regions

To understand the genotype–phenotype associations of WSS, we reviewed the WSS patients previously reported in literature and HGMD databases, and compared the clinical manifestations of those patients sharing the same mutated protein regions with our patients (Table 1). A total of 16 variants, including three variants (p.Glu219Leufs*27, p.Arg301*, and p.Pro280Thr) in the AT‐hook domain, five frame‐shift variants (p.Ser1299Profs*2, p.Gly1338Valfs*18, p.Val1347Trpfs*9, p.Pro1281Leufs*75, and p.Pro1354Leufs*2) and a splicing variant (c.4086+1G>A) in the intermediate region between CXXC and PHD domains, as well as seven missense variants (p.Arg1154Trp, p.Cys1155Tyr, p.Cys1161Gly, p.Gly1168Asp, p.G1181Asp, p.Cys1189Tyr, and p.Cys1194Tyr) in the CXXC domain of KMT2A protein, had been reported to be causative in WSS patients. Given that the phenotype of a patient with variant c.4086+1G>A was not available, we excluded this patient from the following phenotypic comparisons.

Among the patients carrying 15 known and 3 novel variants, none of them had consistent manifestations (Table 1). Nevertheless, some typical WSS clinical signs, such as intellectual disability (17/17; 100%), short stature (17/18; 94.44%), development delay (14/16; 87.5%), hypertelorism (14/18; 77.78%), hypertrichosis (13/18; 72.22%), and feeding difficulties (12/17; 70.59%), were generally present in these patients (Table 1).

When assessing symptomatic severity among three groups of the patients, we found that the patients with mutated CXXC domains were in the most severe forms, followed by those with mutated intermediate region between CXXC and PHD domains and then those with mutated AT‐hook domain. Evident symptoms of the eight patients with mutated CXXC domains included short stature (8/8; 100%), microcephaly (5/8; 62.5%), hypertelorism (7/8; 87.5%), wide nasal bridge (7/8; 87.5%), thin upper lip (7/8; 87.5%), down‐turned corners of the mouth (5/5; 100%), muscular hypotonia (5/5; 100%), thick eyebrows (6/8; 75%), long eyelashes (6/8; 75%), hypertrichosis (8/8; 100%), epilepsy (3/6; 50%), developmental delay (6/6; 100%), language delay (7/7; 100%), intellectual disability (8/8; 100%), behavioral issues (3/7; 42.86%), abnormal brain MRI (3/3; 100%), and feeding difficulties (6/8; 75%). In contrast, three of these features, that is, microcephaly (1/7; 14.29%), epilepsy (1/7; 14.29%), and behavioral issues (1/7; 14.29%), were not evident in the patients with mutated intermediate region between CXXC and PHD domains, while nine features of microcephaly (0/3), wide nasal bridge (1/3; 33.33%), thin upper lip (0/3), thick hair (1/3; 33.33%), long eyelashes (0/3), epilepsy (0/3), language delay (0/3), abnormal brain MRI (1/3; 33.33%), and feeding difficulties (1/3; 33.33%) were not evident in the patients with mutated AT‐hook domain.

## 4 DISCUSSION

We described three patients with neurodevelopment delay together with multiple malformations and identified three novel *de novo* causative variants in *KMT2A*, a gene known to be responsible for WSS. In line with WSS‐related reports, the three patients exhibited representative characteristics of WSS, such as short stature, developmental delay, mental retardation, hypertrichosis, and dysmorphic facial features. They also had some other distinctive symptoms which were sporadically observed. Baer et al. reported that eye anomalies were present in 59% of their studied WSS cohort, while the co‐occurrence of ptosis and astigmatism were found in only one patient (Baer et al., 2018). Notably, our patient 1 had severe ophthalmological symptoms of epicanthus tarsalis (a type of epicanthic fold), upper eyelid ptosis, small palpebral fissures, and astigmatism. It was reported that 35.44~36% WSS patients had cardiac defects, including 28.89% patent ductus arteriosus, and 8.89% patent foramen ovale (http://www.wssfoundation.org/), but WSS case implicated with endocardial fibroelastosis has not been previously reported. In this study, we found that patient 2 presented congenital heart defects of endocardial fibroelastosis, patent ductus arteriosus, and patent foramen ovale. Furthermore, we found that patient 2 had cytomegalovirus infection after birth, which were probably due to prenatal cytomegalovirus infection or congenital immunodeficiency. Although, WSS cases with congenital immunodeficiency had been previously reported, and their main manifestations were recurrent infections of respiratory tract or urinary tract, and significantly reduced immunoglobulin level (IgG, IgA, and IgM) in peripheral blood (Bogaert et al., 2017; Stellacci et al., 2016), patient 2 had normal immunoglobulin and complement levels which were described in detail above. Patient 3 had feet eversion, which has not, to the best of our knowledge, been reported, although puffy or small feet were reported to be in frequency of 22% in WSS cases (Enokizono et al., 2017). Therefore, variants in KMT2A cause a wide spectrum of clinical phenotypes, and WSS is a complex multi‐system disorder that might be sometimes difficult for the clinical diagnosis.

Endocardial fibroelastosis is a thickening of the endocardium due to layers of collagenous and elastic fibers. It might result from viral infection, inflammation, autoimmunity, metabolic anomalies, developmental defects, and genetic factors (Lurie, 2010). For example, genes (e.g., *TAZ*, *NEX*, *MYH7*, and *CSRP3*) have been demonstrated to involve the development of endocardial fibroelastosis (Aherrahrou et al., 2016; Brady et al., 2006). Given that our patient 2 had cytomegalovirus infection and genetic defects, and WSS is a multi‐systematic anomaly, we suggest that further research on the etiology of endocardial fibroelastosis in WSS cases is necessary in the future.

WSS patients associated with mutated AT‐hook (amino acids 169–180, 217–227, and 301–309) of KMT2A are yet limited. p.Lys295* identified in our patient 1 is the fourth variant identified in AT‐hook. Two truncating variants p.Glu219Leufs*27 (at AT‐hook 2) and p.Arg301* (at AT‐hook 3), as well as a missense variant p.Pro280Thr (between AT hook 2 and AT‐hook 3) have been previously reported (Baer et al., 2018; Li et al., 2018; Miyake et al., 2016). Although hypertelorism, developmental delay, and intellectual disability are found in at least two of these four patients, our phenotypic comparison reveals that the patients with mutated AT hooks still lack representative or consistent features. There are three possible explanations for these observations: (i) the number of identified variants is small. (ii) the identified patients in this group are too small to show all the malformations. (iii) the impact of AT‐hook variants may be relatively milder than that of the other two groups of variants studied.

Precise function of the intermediate region between CXXC and PHD domain of KMT2A remains poorly understood. Xu et al. reported that CXXC domain could specifically recognize unmethylated CpG dsDNA (Xu et al., 2018). Wang et al. found that PHD fingers are capable of recognition of different histone marks, DNA binding, mediation of protein–protein interactions, and E3 ubiquitin ligase (Wang et al., 2012). Generally, the patients (including six reported patients and our patient 2) with mutated intermediate region between CXXC and PHD domain have more severe symptoms than those carrying variants in AT‐hooks (Baer et al., 2018; Li et al., 2018; Strom et al., 2014). Among these seven patients, some symptoms (such as developmental delay, intellectual disability, craniofacial features of prominent forehead, hypertelorism, deformed palpebral fissures, malformed nasal bridge, and thin upper lip) are universally noticed, and moreover, some symptoms (such as thick eyebrow, long eyelashes, and hypertrichosis) are found to be in high frequency. Of note, one patient with p.Ser1299Profs*2 was found to have epilepsy, which is not frequently observed in the WSS patients (Chan et al., 2019). Accordingly, we speculate that the intermediate region between CXXC and PHD domain affect normal functions of the two domains during the development of multiple systems.

The majority of KMT2A variants associated with WSS have been truncating variants (Jones et al., 2012; Min Ko et al., 2017). However, missense variants are known to explain a significant fraction of the WSS patients observed. In our patient 3, we identified a novel likely pathogenic variant resulting in an amino acid change from cysteine to arginine (p.Cys1167Arg) in the CXXC domain, a domain having the largest cluster of missense variants in KMT2A (Chan et al., 2019). Previously, seven missense variants in CXXC domain have been reported (Baer et al., 2018; Lebrun et al., 2018; Min Ko et al., 2017; Stellacci et al., 2016). Among them, c.3460C>T (p.Arg1154Trp) was found to lead to significantly increased gene expression level, but not change the protein expression level (Lebrun et al., 2018), underlying the importance of CXXC domain. In accordance with the key regulation role of CXXC domain and severe neuro‐phenotypes associated with the variants in this domain (Lebrun et al., 2018; Li et al., 2018; Min Ko et al., 2017), the WSS patients (seven reported patients and our patient 3) with mutated CXXC domain have more severe and profound anomalies than those with mutated AT hooks or intermediate region between CXXC domain and PHD domain (Table 1). Microcephaly (5/8 patients) as well as small hands and feet (6/8 patients) are prevalent in patients with mutated CXXC domain. In terms of neurodevelopment, language delay is presented in 7/7 patients, epilepsy detected in 3/6 patients, aggressive behavior exhibited in 3/7 patients and high severity level of intellectual disability found in 8/8 patients.

In summary, our study would aid in molecular diagnosis of WSS and expand our knowledge about genotype–phenotype correlation of this disorder. WSS heterogeneities in both genotype and phenotype are evident in our patients herein described and in previous reported patients. Given that KMT2A variants is associated with multi‐systematic anomalies, especially in heart, lung, brain, and immune systems, we recommend that multiple tests of spine MRI and CT, immunological examination, growth hormone determination, eye examination, ENT examination, cardiac ultrasound, abdominal ultrasound, EEG and cranial MRI examination, and genetic testing are helpful for assisting clinical diagnosis and management of the WSS patients.

## CONFLICT OF INTEREST

The authors declare no conflict of interest.

## AUTHOR CONTRIBUTIONS

XH and LY conceived the study; SL, BB, YH, and XH clinically analyzed the patients; WZ and PZ conducted genetic data interpretation; WZ and XH performed literature review and prepared the manuscript; WZ, RZ, WC, and XH revised the manuscript. All authors reviewed and approved the manuscript.

## Data Availability

The data used to support the findings of this study are included within the article.
